# Geothermal energy appraisal and subsurface structural mapping of the Rafin Rewa warm spring region, Precambrian basement complex of Nigeria

**DOI:** 10.1038/s41598-024-66927-9

**Published:** 2024-07-29

**Authors:** Ayatu Ojonugwa Usman, Ema Michael Abraham, Churchill Chukwunonso Oknonkwo, Augustine Ifeanyi Chinwuko, George-Best Azuoko

**Affiliations:** 1 Applied Geophysics Program, Alex Ekwueme Federal University, Ikwo, Ebonyi Nigeria; 2 Department of Physics, Federal College of Technology, Umunze, Nigeria; 3 Department of Applied Geophysics, Nnamdi Azikiwe University, Awka, Nigeria

**Keywords:** Warm spring, Geothermal energy, Rafin Rewa, Structural modelling, Magnetic method, Environmental sciences, Energy science and technology

## Abstract

This research work aims at evaluating the geothermal energy potentials of the Rafin Rewa warm spring (RRWS) of the Precambrian Basement Complex in Nigeria as an alternative energy source using integrated aeromagnetic geophysical techniques. Four aeromagnetic dataset were acquired, assemblage, analyzed, and interpreted using integrated geophysical processing techniques of spectral analysis and Euler deconvolution. Qualitative interpretation of the residual anomalous map reveals a distribution of positive anomalies (> 53 nT) majorly in the central and southeastern regions, which are traced to the granitic rocks, while the low anomalies (< − 1.5 nT) have been traced to the RRWS location emanating from the coastal plain sands of the Pliocene, Pleistocene, Oligocene, and Miocene ages. Quantitatively, the depth to the top (DTT) of the anomalous bodies reveals a depression that is almost intersecting with the Curie point depth (CPD) plot at the RRWS location, which indicates high heat flow in the RRWS region. The Spectral Analysis results reveal that the DTT and the CPD in this area ranges from 0.512 to 0.761 km and 6.504 to 10.582 km, respectively while the average CPD is 8.543 ± 0.325 km. It is observed that the DTT and CPD decrease as one move away from the RRWS region. The computed heat flow average was 160.76 ± 19.09 mW/m^2^ within the RRWS region. The Euler deconvolution result reveals the presence of geological structures, which were interpreted as faults and fractures. The major fractures trend in the east–west (E-W) directions, while the minor fractures trend northeast-southwest (NE-SW) directions. The geochemical result presented shows that iconic compositions impact the convective heat transfer processes associated with geothermal systems. It was conclusively believed that regions with comparable shallow CPDs could be viable for further geothermal energy investigations.

## Introduction

Nigeria is currently faced with a challenging energy situation whereby the demand for electric power surpasses its supply. As a result, the country urgently requires alternative sources of energy, preferably renewable sources. The inadequacy of electricity, which is the primary source of energy in Nigeria, has negatively impacted the country's growth and development. In 2013, the Nigerian Federal Government privatized six electric generation and eleven distribution companies. Despite these efforts, the average power generation as of December 2013 was 3800 MW, and in 2014, 3900 MW was produced for a population of over 170 million^1,2^. This insufficient supply of electricity still persists, despite the evident increase in the country's population. Therefore, there's a need to evaluate the available data with a view to exploring alternative means of energy supply. Furthermore, the estimations made by energy operators in the country indicate a necessary investment of $100 billion over 20 years in order to sustain the power deficit^3^ According to the research conducted by^4^ and^5^ substantial investment is necessary in the Nigerian power sector to achieve reliable and suitable power supply, but the current economic conditions in the country render funding for such a project unavailable.

The overarching aim of Nigeria is to establish a secure, abundant, cost-effective, and environmentally sustainable energy supply, primarily attributed to the country's substantial geothermal reserves^4,6,7^. Geothermal gradient, denoting the rate of temperature increase per unit depth in the subsurface of the earth resulting from its core's heat emissions^8–10^, is a widely recognized parameter utilized in geophysical logging. It serves as an indicator of subsurface temperature distribution, aiding in the assessment of an area's geothermal potential and enhancing the understanding of regional and sub-regional tectonics^11–14^. Studies suggest that the average temperature gradient between the earth's core and the outermost limits of its atmosphere is around 100 °C/km. Knowledge about geothermal gradients and heat flow within a location is beneficial in advancing geothermal energy production. Crustal thermal energy associated with hot springs is a phenomenon that occurs through two distinct processes, depending on whether such hot springs are situated in volcanic or non-volcanic regions^11,15,16^.

Rafin Rewa warm spring (RRWS) is situated in the Precambrian complex of Nigeria; the RRWS is characteristically perennial and possesses a maximum flow rate of 0.1 L/s. The temperature of water emanating from the springs is routinely measured at 42.2 °C^17^. The likely source of RRWS can be traced to recent magmatic activity that has occurred within the Jos Plateau region of Nigeria since the Tertiary period.

**Figure 1:**
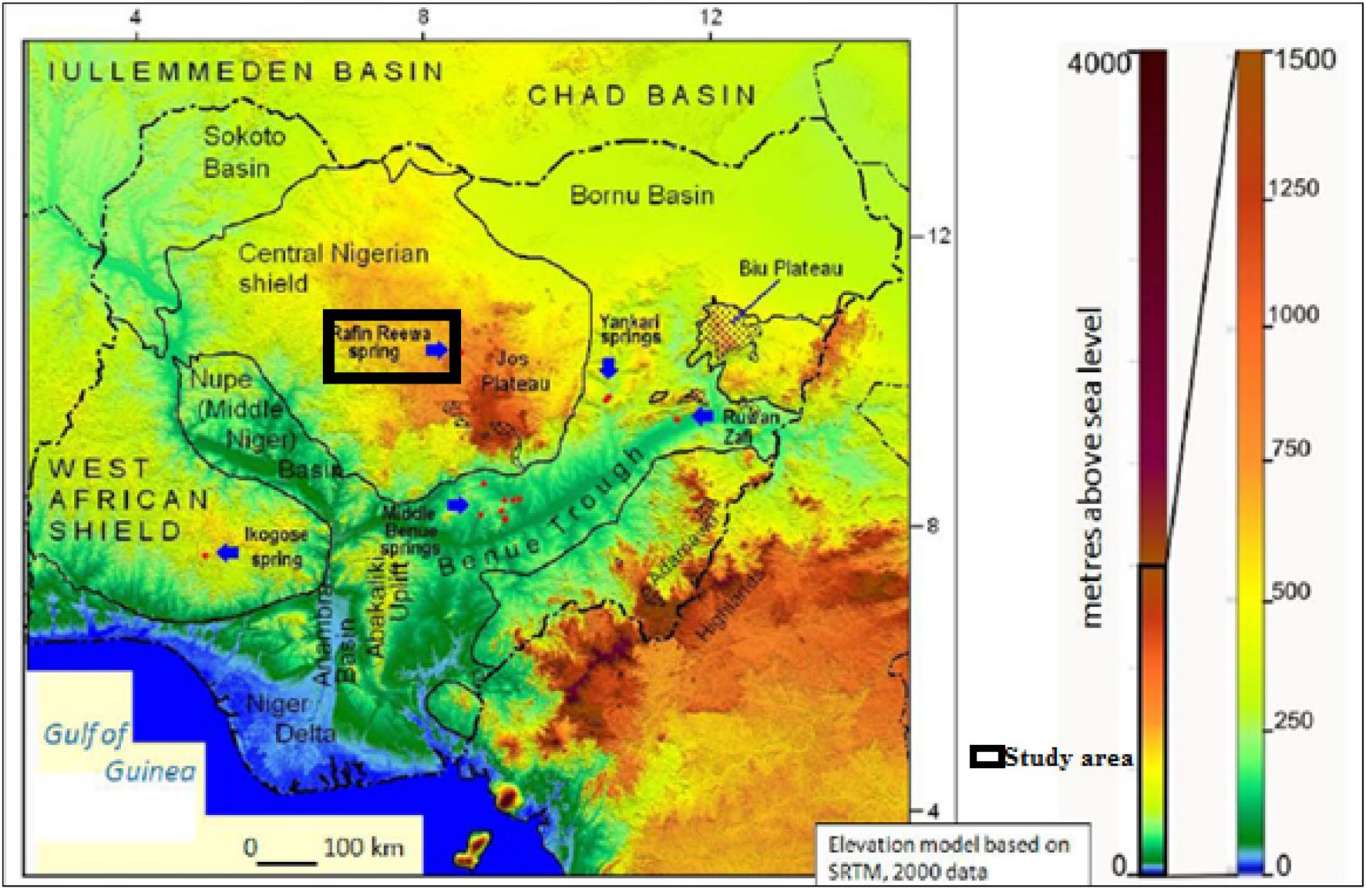
Location map of the study area over Nigeria geothermal Hot Spring. Map generated from STRM 900 Digital Elevation database v4.1 (https://csidotinfo.wordpress.com/data/srtm-90m-digital-elevation-database-v4-1/).

The present study constitutes a pioneering attempt to assess the potential for geothermal energy in the Rafin Rewa Warm Spring (RRWS) region, which has not yet been investigated. With an impressive average surface temperature of 42.2 °C, RRWS ranks as one of the two hot springs in Nigeria with high temperature values. Previous research focused on warm springs, with surface temperatures ranging between 30 and 54 °C, in hot springs such as Warri and Akiri^18,19^ (Fig. 1). The prospect of geothermal exploration in this area is highly encouraging. To this end, we will be conducting a comprehensive appraisal of the region and its environs, utilizing an integrated suite of magnetic geophysical techniques, including Euler deconvolution, spectral analysis (SA), and analytical signaling (AS). These techniques will help to appraise the geothermal potential of the research region. The integrated model of our findings will aid in assessing the basin geomorphology that is emanating the thermal heat. Additionally, the integrated model of the Curie depth point (CDP) and Euler structural model results of the area will facilitate accurate incorporation and identification of productive zones for potential drilling and recommendations aimed at bolstering Nigeria's geothermal energy development in line with the short-term objectives outlined in Nigeria's Renewable Energy Master Plan, as well as future contributions towards renewable energy supply.

### Geology of the researched region

**Figure 2:**
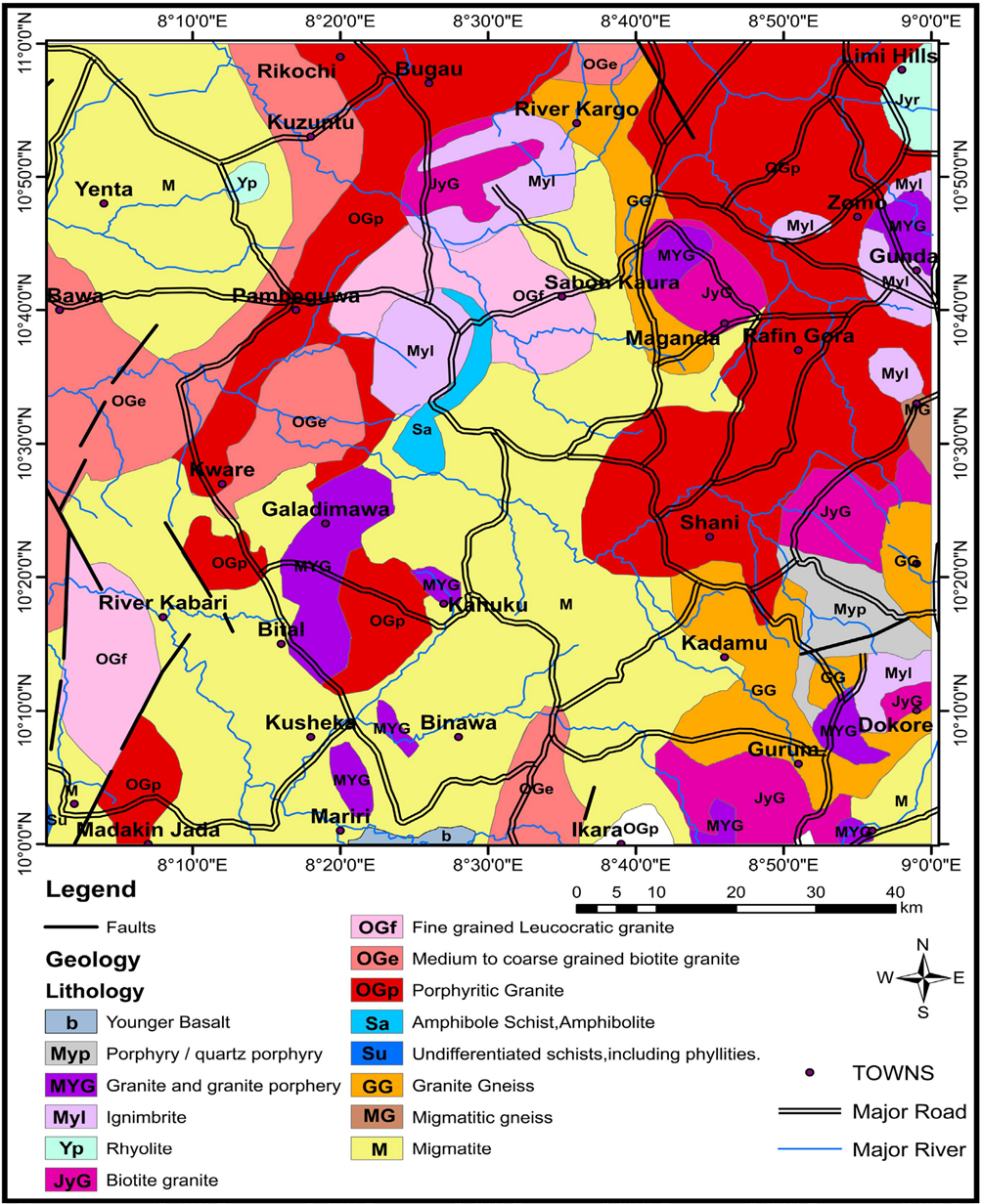
Local Geology map of the research region, Map generated using ArcGIS 10.8 software (https://desktop.arcgis.com/en/arcmap/latest/get-started/setup/arcgis-desktop-system-requirements.htm).

The research region is situated in central Nigeria, specifically on the southwest edge of the Jos Plateau adjacent to the Lisiwa ring complex (Fig. 2). The region is classified as the young granite of the Jurassic era, with granites from the "young granite" series appearing mostly as ring complexes consisting of sodalite, amphibole, biotite, linear granite, syenite, and trachyte, and smaller gabbro and dolerite, with rhyolite, clusters, and ignimbrite rarely preserved (Fig. 2). The centers commonly overlap, with intrusions generally moving towards the south. However, there are no evident superficial connections between the site and regional crustal structures, even though the northeasterly trend of the complex is apparent, possibly indicating underlying vulnerabilities in the basement rocks^20^. Obaje^21^ have noted a series of hydrothermal alteration processes along with corresponding mineralization within the anorogenic ring.

The research area is a constituent part of the extensive pan-African orogeny that impacted the southern region of Nigeria during the late Proterozoic and early Paleozoic eras. The igneous rock is composed chiefly of plagioclase mica, quartz and feldspar, with marginal quantities of amphibole, biotite, and hornblende. When it is fresh, it appears to be gray to pink in color but changes to a deep red or purple tint when it is exposed to weathering^22^. The Younger granite is thought to have been generated during the Pan-African orogeny, which is believed to have been caused by the merging of the Congo and West African cratons. Due to this merging, the rocks of the Jos Plateau were subjected to high temperatures and pressures, eventually melting and turning into the Younger granite. The younger granite can be found in various spots throughout the plateau, including outcrops in Jos, Bukuru, and Kanam. Its endurance and long-lasting qualities make it a desirable building material in the region, due to its durability and long-lasting properties. It has also been used for the construction of roads, monuments, and even some of the area’s unique rock formations.

The geology of RRWS and its surrounding region is characterized by igneous, metamorphic, and sedimentary rocks that are part of the Precambrian basement complex in Nigeria^17,23^. This intricate geological composition gives way to various warm springs and hydrothermal deposits that hold paramount importance in providing fresh water to the local environment. Furthermore, the geology of the area has significant implications for the potential of regional geothermal energy. Thus, a comprehensive examination of rock types, structures, and hydrothermal features is imperative to fully comprehending the geology of this locale.

## Methodology

Four High Resolution Aeromagnetic Data (HRAM) sets, specifically sheets 126 Dutsen Wai, 126 Ririwai, 146 Bital, and 147 Rahama, were the input data used in this work. The data was acquired from the Nigerian Geological Survey Agency in Abuja (NGSA) and had already been converted to digital form, with a line spacing of 500 m along a flight line spanning 2 km. Prior to the utilization of the HRAM, the ambient magnetic field and diurnal magnetic effects were removed from the data. The HRAM data sets were integrated and contoured to produce the Total Magnetic Intensity (TMI) anomalous map. This TMI anomalous map reflected both the residual and regional anomalous field components. A straight trend surface was fitted into the data through multiple regression techniques^24^ to separate the two fields and enable interpretation of the local bodies.

Integrated geophysical techniques were utilized to interpret and model the local anomalies from the data. It is worthy to note that no single geophysical method can provide conclusive results or recommendations; thus, the combination of different geophysical techniques is essential to increase the confidence level in the interpretation. In this particular study, spectral analysis and Euler deconvolution techniques were integrated into the thermal depth calculations and subsurface structural modeling. The spectral analysis technique involved the use of digitized residual anomalous map (RAM) data, which was then converted into a Fourier domain to compute the energy spectrum. The logarithmic scale of amplitude against frequency was plotted, and by analyzing the slopes of the segments, the estimates of average depths for magnetic sources of anomalies in the study region were determined. Furthermore, by dividing the slope of the computed wavelength signal of the outward average of the individual power spectrum by the radial frequency, more accurate and precise results were obtained.

The mathematical background of the Spectral analysis was summarized by^25^ and shown below:

Fourier Transform analysis summarized by^26^ and shown below:

1 $${Y}_{i\left(x\right)=}\sum_{n=1}^{N}\left[{a}_{n }\text{cos}\left(\frac{2\pi n{x}_{i}}{L}\right)+{b}_{n }\text{sin}\left(\frac{2\pi n{x}_{i}}{L}\right)\right]$$

where: ${Y}_{i}\left(x\right)$ = Reading at ${x}_{i}$ position, [L = cross-sectional length of the anomaly, n = partial wave harmonic number, N = data points number—Partial amplitude], ${a}_{n}$= amplitude spectrum real part, ${b}_{n}$= amplitude spectrum imaginary part, i = 0, 1, 2, 3,…, n

and,

2 $${{a}_{n}}_{ = \frac{2}{N}} \sum_{i=1}^{N}{Y}_{i}\text{cos}\frac{2\pi n{x}_{i}}{L}$$

3 $${}_{{b}_{n} = \frac{2}{N}} \sum_{i=1}^{N}{Y}_{i} \text{sin}\frac{2\pi n{x}_{i}}{L}$$

The work of Ikumbur et al.^15^ shows the main amplitude signal (A_n_) as:

4 $$ A_{n} = \sqrt {a_{n}^{2} + b_{n}^{2} } $$

A graph of A_n_ again frequency (n) was then plotted in the natural log graph and a linear trend of the low-frequency signal of the spectrum was plotted from the result and it shows the attributes of the deep-lying anomalous body. The gradient of the straight segment were analyzed and the depth to the subsurface anomalous body was calculated using the model developed by^27,28^:

5 $$z = -ML/2\pi $$

where, $z$ = basal depth; $M$ = slope of the linear section; $L$ = anomalous body width.

According to^10,29^ Fourier transform methods are particularly useful in spatial data analysis such as the magnetic data. This present research focuses in appraising the geothermal energy potential of the RRWS region from the depth calculation and modelling of the basal depth of the magnetic bodies (Curie point depth) and heat flow around the region. The gradient of the computed wavelength signal of the outward average of the individual power spectrum was divided by the radial frequency to give depth to the center (centroid) of the anomalous deepest basal block (Zo). The depth to top (Zt) of the anomalous body was evaluated from the gradient of her second-longest wavelength part of the spectrum^30–32^. A geothermal gradient model map and a 3D subsurface heat flow map were also generated from the calculated depth parameter values stated above.

6 $$\text{In}\left[\frac{\uprho (\sqrt{\text{S}})}{/\text{S}/}\right]=\text{InA}-2\uppi /\text{S}/{\text{Z}}_{\text{o}}$$

ρ(s) is the radial average of the power, Z_0_ is depth to the centroid, A = constant^6^.

Biswas and Odoh et al.^33,34^ model was used to calculate the depth to the top (DTT) of the anomalous body (Zt).

7 $$In\left[\frac{\rho (\sqrt{S})}{/S/}\right]=InB-2\pi /S/{Z}_{t}$$

B = sum of K without the gradient^35^

Equation (8)^36^ was deployed in calculating the depth to the bottom of the anomalous bodies.

8 $${Z}_{b}=2{Z}_{o}-{Z}_{t}$$

From the works of^17,31,36^ the depth to bottom estimated is also the Curie point depth.

The heat flux density and thermal flow density for the region were estimated using^37,38^ model (Eq. 9).

9 $$\text{q}=\uplambda \frac{dT}{dZ}$$

q = heat flux density and λ is the transferred heat coefficient^14,17^.

The work of^39^ was used to calculate the temperature gradient (Eq. 6).

10 $$\uptheta = \left[\frac{dT}{dZ}\right]{Z}_{b}$$

The 3-D Euler's homogeneity equation, which establishes a relationship between the field's magnetic field and its gradient components and the location of the sources, is determined by the amount of homogeneity N. This is determined as a structural index^37^. The method in question uses a structural index for estimating depth. The structural index and depth estimations can be used to detect and calculate depth for a variety of geological formations, including faults, magnetic contacts, dykes, sills, and more. A recorded solution is only valid if the uncertainty of the calculated depth estimate is less than a predefined threshold and the location is within the data window's center limit distance. The study also utilized the^5,26^ models in subsurface structural modeling in the region. This was further integrated with the spectral analysis result to appraise the geothermal energy potential of the region.

### Ethics declarations

The authors adhere to all ethical standards in the analysis and draft manuscript writing.

## Results

**Figure 3:**
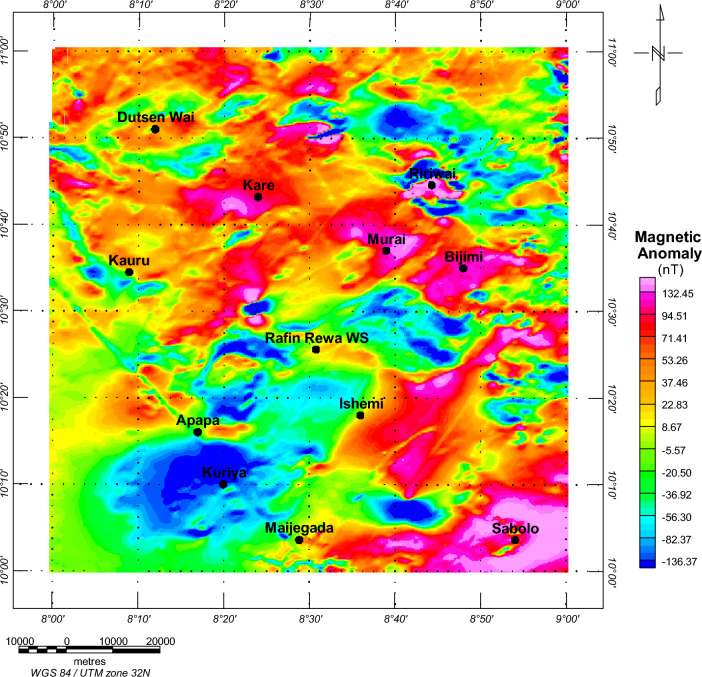
Residual magnetic anomalies at the Rafin Rewa Warm Spring region (RRWS). Map generated using Oasis Montaj Software 9.3 (https://www.geometrics.com/software/geosoft-oasis-montaj/).

Qualitative interpretation was done by visually examining the total magnetic intensity (TMI) and residual anomalous map (RAM) (Fig. 3). High magnetic intensity was observed in the central and southeastern parts of the research area (specifically Kare, Murai Bijimi, and Kauru regions), whereas low magnetic intensity values are present in the western, northern, and central regions (including Rafin Rewa region) (Fig. 3). The RAM illustrates a general contour trend from northeast to southwest (Fig. 3). Both the southern and northern regions of the research area exhibit high magnetism, with findings from^9,40^ suggesting a magnetic contrast in faulted or fractured zones due to processes like magnetite oxidation to hematite or the filling of fracture regions with vein deposits.

Upon conducting a visual assessment of the anomalous RAM map within the research area, it was observed that the central regions (Rafin Rewa regions) exhibit low magnetic intensity values which ranges from − 5.57 to − 82.3 nT, indicating the presence of thick sediments infilling the area. Conversely, areas such as Kare, Murai Bijimi, and Kauru show high magnetic intensity values ranging from 53.26 to 132.45 nT, suggesting a thin sedimentary layer. Additionally, the Ishemi area, situated to the northwest of the Rafin Rewa region, displays high magnetic intensity values, implying a shallower sedimentary pile. The structural characteristics of these interconnected regions indicate the potential for geothermal energy migration, as illustrated in Fig. 3.

### Quantitative interpretation

**Figure 4:**
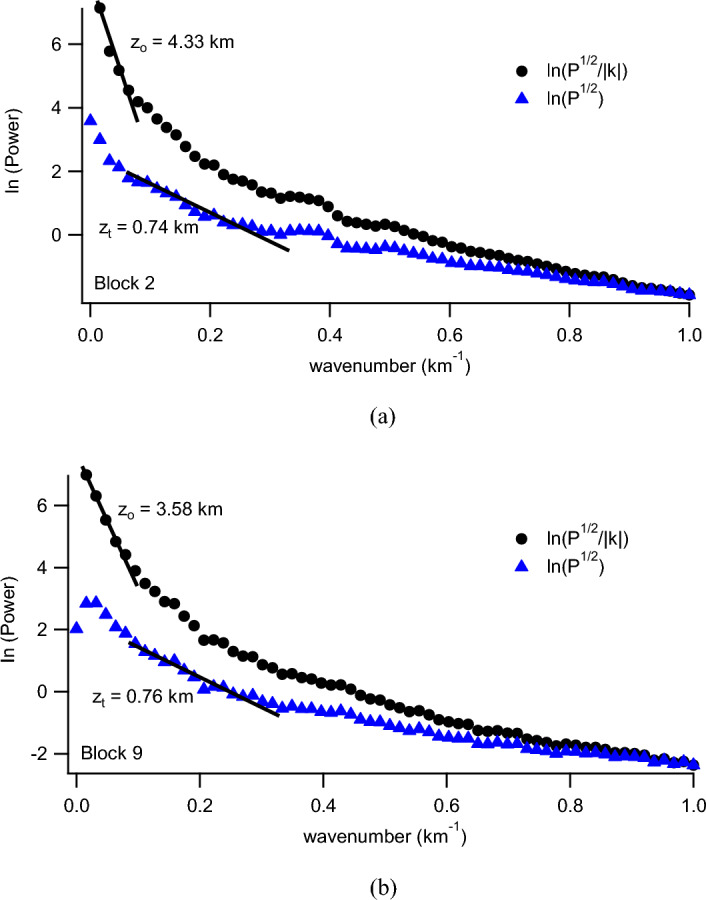
Sample spectral plots for radially average power spectrum computations (Blocks 2 and 9). Data from these spectra, derived from the windows analyzed, were used for the estimation of the CPD and subsequent heat flow computations. Generated using Oasis Montaj Software 9.3 (https://www.geometrics.com/software/geosoft-oasis-montaj/).

Quantitative interpretation serves the primary purpose of calculating depth and modeling anomalous bodies. Geophysical techniques such as spectrum analysis and Euler deconvolution were used in this study to calculate depth and model the subsurface geologic structures. The study region was separated into spectrum overlapping pieces of 37 km^2^. The energy spectrum was generated by conducting spectral analysis on each individual blocks (Fig. 4). The energy spectrum shows that the deeper sources are represented at the lower wavenumber end, whilst the shallower ensemble appears at the higher wavenumber. The tail end of the spectrum is primarily due to noise at higher wavenumbers. The depth computations were performed using the Fast Fourier Transform filter, which transferred the data from the spatial domain to the wavenumber domain. The wavenumber increase in the final transform is calculated as 1/(line length).

**Figure 5:**
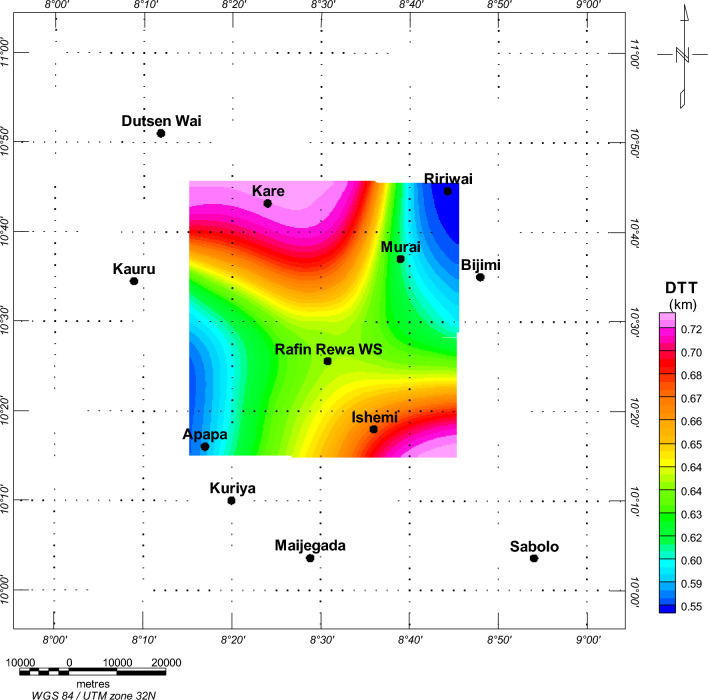
DTT of magnetic sources within the RRWS region. The depths increase away from the RRWS to the northern and southeastern regions of the RRWS region. Map generated using Oasis Montaj Software 9.3 (https://www.geometrics.com/software/geosoft-oasis-montaj/).

**Figure 6:**
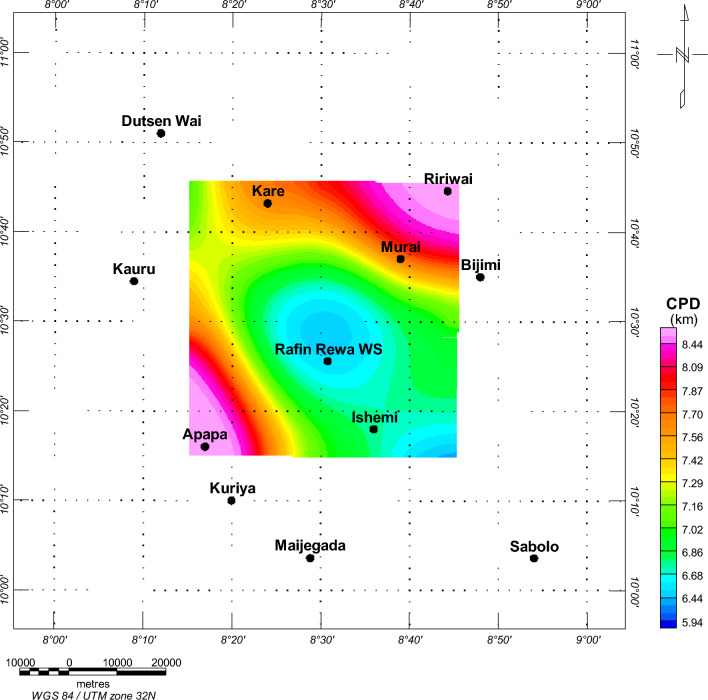
CPD results derived from the spectral analysis of aeromagnetic data from the region. The RRWS region indicates shallow CPD values between 6.44 and 6.6 km. The CPDs appear to increase away from the RRWS region. Map generated using Oasis Montaj Software 9.3 (https://www.geometrics.com/software/geosoft-oasis-montaj/).

**Figure 7:**
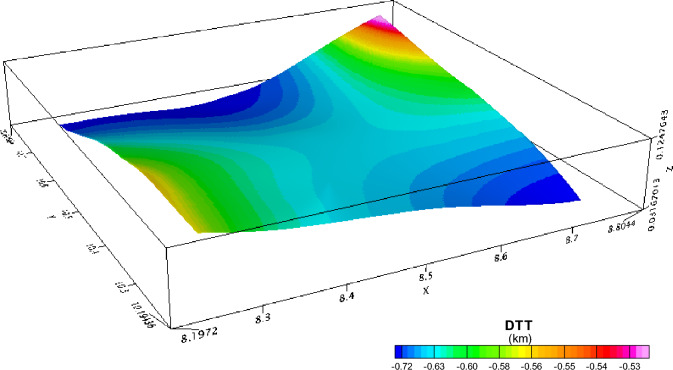
3D representation of the DTT of magnetic sources. Generated using Oasis Montaj Software 9.3 (https://www.geometrics.com/software/geosoft-oasis-montaj/).

**Figure 8:**
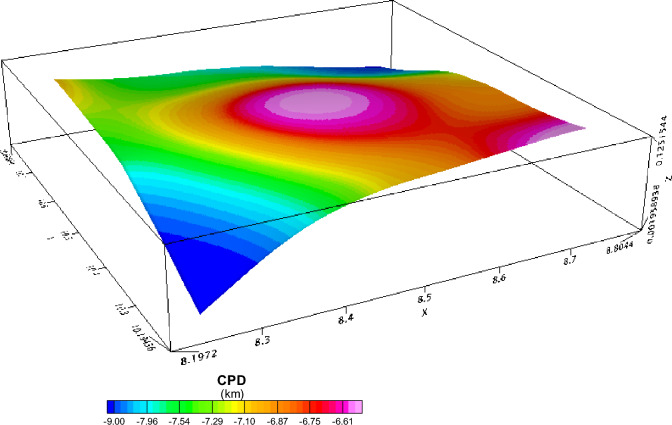
3D representation of the estimated CPD in RRWS region. Generated using Oasis Montaj Software 9.3 (https://www.geometrics.com/software/geosoft-oasis-montaj/).

**Figure 9:**
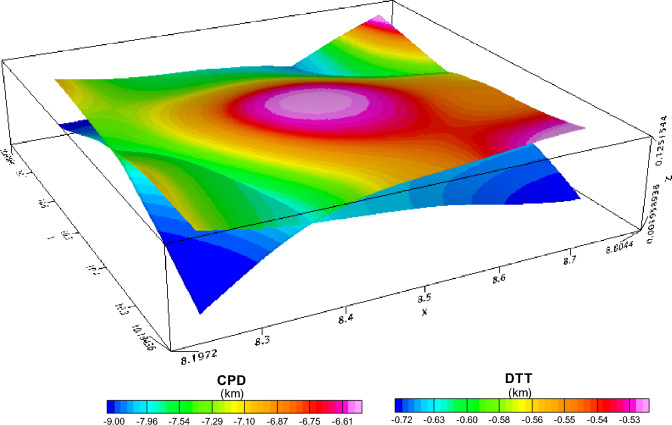
A combined representation of the DTT and CPD estimations from analysis of magnetic data from the RRWS region. Generated using Oasis Montaj Software 9.3 (https://www.geometrics.com/software/geosoft-oasis-montaj/).

An investigation was conducted on the Depth to the Top (DTT) and Curie Point Depth (CPD) for the Rafin Rewa warm spring region, as presented in Table 1, Figs. 5, and 6. The DTT and CPD in this area range from 0.512 to 0.761 km and 6.504 to 10.582 km, respectively. The average CPD is 8.543 ± 0.325 km. It is observed that both the CPD and DTT decrease as one moves away from the Rafin Rewa warm spring region. The centroid depth averages 4.574 ± 0.042 km. Additionally, the 3D model illustrates that the sedimentary layer thins away from the Rafin Rewa warm spring region, displaying an undulating rather than a horizontal structure (Fig. 9). Furthermore, the 3D CPD model highlights that the sedimentary infillings and basal depth of the magnetic layer are structurally controlled, as depicted in Figs. 7 and 8.

**Table 1:** Results of spectral analysis computations and depth estimations of magnetic sources. Their accompanying error estimations are also presented.

Longitude	Latitude	Location name	z_t_ (km)	Error z_t_ (± km)	z_o_(km)	Error z_o_ (± km)	CPD (km)	Error CPD (± km)
8.25	10.75	Bugau	0.738	0.022	3.884	0.045	7.031	0.372
8.50	10.75	Zomo	0.741	0.023	4.329	0.023	7.916	0.331
8.75	10.75	Muria	0.512	0.016	4.836	0.029	9.160	0.387
8.75	10.50	Kare	0.599	0.012	3.847	0.070	7.095	0.400
8.50	10.50	Bijimi	0.641	0.007	3.573	0.055	6.504	0.267
8.25	10.50	Rafin Rewa	0.593	0.011	4.113	0.023	7.633	0.230
8.25	10.25	Galadimawa	0.568	0.015	5.575	0.029	10.582	0.388
8.50	10.25	Sabolo	0.653	0.022	3.856	0.033	7.058	0.354
8.75	10.25	Ungwwar Gulawa	0.761	0.014	3.586	0.032	6.412	0.230

**Figure 10:**
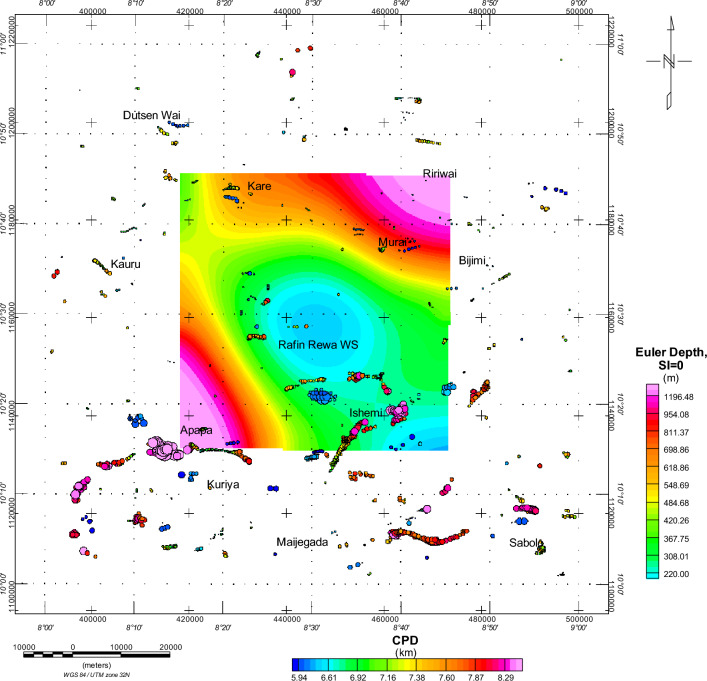
Euler depth results on the CPD map. The structural index SI = 0 investigates the existence of notable geological contacts (faults or fractures) in the region. Generated using Oasis Montaj Software 9.3 (https://www.geometrics.com/software/geosoft-oasis-montaj/).

**Figure 11:**
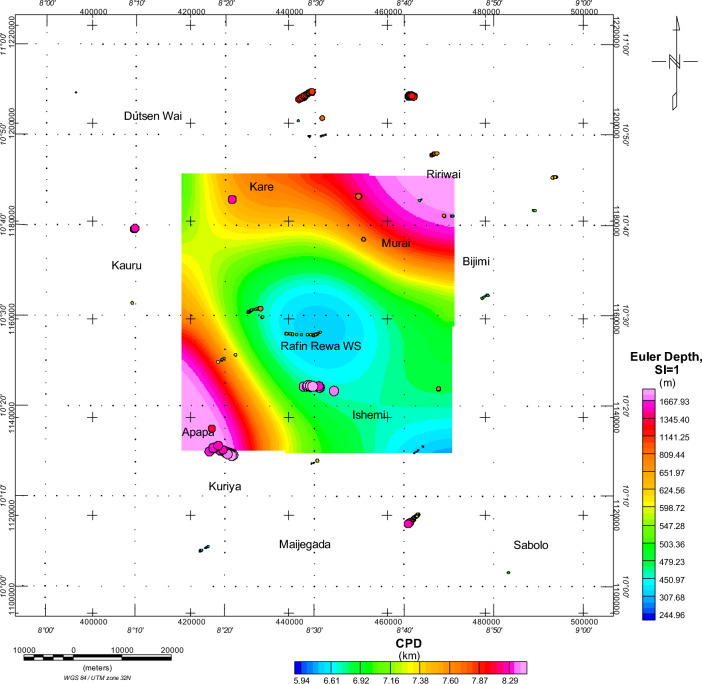
Euler depth results on the CPD map. The structural index SI = 1 investigates the existence of notable geological intrusions in the region. Generated using Oasis Montaj Software 9.3 (https://www.geometrics.com/software/geosoft-oasis-montaj/).

Within our research, the Euler deconvolution technique using a structural index (SI) of both 0 and 1 was used in mapping the predominant subsurface structures (Fig. 9). This approach successfully helped us identify and map the sill and dyke structures in the study area. Using SI of 0 (Fig. 10), we unveiled two distinct depth sources: the deeper sources (high sedimentary pill) and the shallower sources (thin sedimentary infilling). The depths of the magnetic sources range from 220 to 1196.48 m, primarily located outside the RRWS region. The subsurface geological structures appear to trend in an east–west direction (E-W). The high solution concentration, which was delineated as dyke, was predominantly situated in the southern portion of the study area, as shown in Fig. 10. Furthermore, the results obtained from the Euler deconvolution analysis using an SI of 1 (Fig. 11) indicated a lower concentration of solutions within the RRWS region, but there is evidence of solution concentration around the Apapa and Ishemi areas showing high structuration and faulting.

## Discussion

The magnetic anomalies (Fig. 3) in the region of this study show a distribution of positive anomalies (> 53 nT), primarily in the central and southeastern regions. These positive anomalies are mostly traced to the granitic rocks of the region (Fig. 2). Low anomalies (< − 1.5 nT) have been traced to the RRWS location emanating from the coastal plain sands of the Pliocene, Pleistocene, Oligocene, and Miocene ages. We believe that the low magnetic anomalies in the region could have been partly influenced by the presence of the heat source at RRWS at the location. Abraham et al. and Usman et al.^6,39^ had earlier observed low magnetic anomalies at a warm spring location with similar geologic characteristics as the RRWS region.

Data computed from Fig. 5 and displayed in Table 1 show depths to the top (DTT) of magnetic sources ranging between 0.51 and 0.76 km. DTT evaluation reveals shallow depths at the RRWS location at an average of 0.63 km and tends to increase away from RRWS at the northern and SE locations. A more shallow DTT (< 0.55 km) is observed in the NE and SE regions of the Ririwai and Apapa regions and traced to the granite formation in these regions. Average CPDs between 6.41 ± 0.23 and 10.58 ± 0.39 km are observed in the study area. Estimated CPDs (Fig. 6) at the RRWS location range between 6.4 and 6.6 km and become deeper towards the NE and SW regions of the study area. The shallow CPD at the RRWS location could be due to subsurface geologic intrusions in the undifferentiated basement complex of the location. We believe that these intrusions may be responsible for the heat source in the region, especially as the CPDs become deeper away from the spring location. Comparative shallow CPDs are also observed southwards of the RRWS along the Ishemi location. This region is located partly on the undifferentiated basement complex and the older granite formations. The basement complex is in places intruded and interspersed with older granites that originated in the Pan-African orogeny^6^. We believe these regions, with comparable shallow CPDs, could be viable for further geothermal energy investigations.

The 3D representation of the estimated depths (Figs. 7, 8, and 9) reveals a clear intrusion within the subsurface, portraying the central region of the study area (RRWS location) as shallow in comparison with the other regions. In addition, a depression of DTT almost intersecting with the CPD plot at the RRWS location (Fig. 9) could signify high heat flow at the RRWS location. This hypothesis is confirmed by the estimated heat flow in the region. The computed heat flows for the study area average 160.76 ± 19.09 mW/m^2^. Values of heat flow in a region in excess of about 80 to 100 mW/m^2^ indicate anomalous geothermal conditions in the subsurface^4,6,7,31^ and are worthy of further investigation. The presence of RRWS at this location further strengthens this deduction.

Examination of the possible influence of geological contacts on the CPD results (Fig. 10) shows minimal surficial influence of geological contacts on the CPDs. Good clustering of solutions from the Euler deconvolution indicates geological contacts that could possibly be interpreted as faults and fractures within the RRWS location at depths ranging from 480 to 618 m. The general trend is in the EW and NE-SW directions. Figure 11 shows possible intrusions that could be reached at depths of 547 and 1700 m within the regions with shallow CPDs. This could also be a reflection of the subsurface intrusions of granitic rocks within the RRWS region. Table 2 shows the results of the geochemical analysis conducted on the RRWS. The pH value of 8.1 indicates that the subsurface fluids are slightly alkaline. This alkalinity could promote the dissolution of certain minerals in the subsurface rocks at the RRWS location. This can affect the porosity and permeability of the rocks, which in turn influences their thermal conductivity and heat transfer properties. The electro-conductivity value of 350 S/cm (Table 2) at the RRWS location also provides insights into the ionic content and salinity of the subsurface fluids. These are important for understanding their thermal behavior and influence on heat transfer processes.

**Table 2:** Results of laboratory analysis of the Rafin Rewa Warm Spring^23^.

Parameter	Samples from Rafin Rewa Analysed by Glowny Instytut Gornictwa, Katowice
PH	8.10
Electro-conductivity	350 µS/cm
Total dissolved solids	214 mg/l
Calcium	1.50 mg/l
Magnesium	0.06 mg/l
Sodium	88.51 mg/l
Potassium	1.64 mg/l

The moderate electro-conductivity value of 350 S/cm suggests that the subsurface fluids in RRWS have a certain level of salinity. Salinity affects the density and heat capacity of the water, which in turn influences its thermal behavior and heat transfer properties^40^. The Total Dissolved Solids (TDS) value of 214 mg/l at RRWS provides insights into the concentration of dissolved substances in the subsurface fluids, which could impact the thermal conductivity and heat capacity of the water. The presence of these dissolved substances affects the heat capacity of the spring. This parameter is important for understanding the amount of heat energy that the water can store and transfer, thus influencing its thermal behavior. The implications of the obtained concentration values of 2.6 mg/s calcium, 4.4 mg/s magnesium (Mg), 0.71 mg/s sodium (Na), and 0.84 mg/l potassium (K) are related to the iconic composition of the fluids, their thermal behavior, and their potential influence on thermal conductivity and heat transfer processes at RRWS. The iconic composition can impact the convective heat transfer processes associated with geothermal systems. These variations in the iconic composition of the analyzed samples (Table 2) from RRWS can influence fluid circulation, heat exchange, and the overall thermal regime of the RRWS region.

## Conclusion

The RRWS region was analyzed using integrated geophysical methods including Spectral Analysis and Euler deconvolution. Qualitative magnetic data suggests a shallow basal depth and increasing magnetism away from RRWS. Sedimentary layers show uneven, undulating features influenced by local structure. Quantitative analysis links heat flow density to subsurface structures, highlighting potential heat flow conduits. A 3D model incorporating DTT and CPD identifies a heat flow depression near RRWS, with CPD averaging 6.41 ± 0.23 and 10.58 ± 0.39 km. Shallow CPDs suggest intrusive activity and high heat flow, potentially aiding geothermal exploration. Heat flow averages 160.76 ± 19.09 mW/m^2^, exceeding typical ranges, prompting further study. These heat flow values exceed the range of 80 to 100 mW/m^2^ and exhibit favorable comparison with previous studies by^4,7,19,31^, warranting further investigation. The presence of the Remote Reservoir Water Signatures (RRWS) at this location provides additional support for this inference. Euler deconvolution reveals faults and fractures at depths of 480–618 m with EW and NE-SW trends. Fluid composition affects convective heat transfer, affirming geothermal potential in the RRWS region.

## Data Availability

The data used in the study is with the corresponding author and he will make it available on reasonable request.

## Abbreviations

RRWS: Rafin Rewa warm spring
DTT: Depth to the top
CPD: Curie point depth
HRAM: High resolution aeromagnetic data
TMI: Total magnetic intensity
RAM: Residual anomalous map
